# Molecular docking and dynamics simulation of FDA approved drugs with the main protease from 2019 novel coronavirus

**DOI:** 10.6026/97320630016236

**Published:** 2020-03-31

**Authors:** Hasanain Abdulhameed Odhar, Salam Waheed Ahjel, Ali A.Mohammed Ali Albeer, Ahmed Fadhil Hashim, Ali Mahmood Rayshan, Suhad Sami Humadi

**Affiliations:** 1 Department of pharmacy, Al-Zahrawi University College, Karbala, Iraq

**Keywords:** 2019-nCoV, main protease, repurposing, docking, dynamics simulation

## Abstract

Design and development of an effective drug to combat the 2019 novel coronavirus remains a challenge. Therefore, it is of interest to study the binding features of 1615 FDA approved drugs with the recently known 2019-nCoV main protease structure having high sequence homology with that from SARS-CoV. We document the binding features of top 10 drugs with the target protein. We further report that Conivaptan and Azelastine are mainly involved in hydrophobic interactions with active site residues. Both drugs can maintain close proximity to the binding pocket of main protease during simulation. However, these data need further in vitro and in vivo evaluation to repurpose these two drugs against 2019-nCoV.

## Background

**Figure 1:**
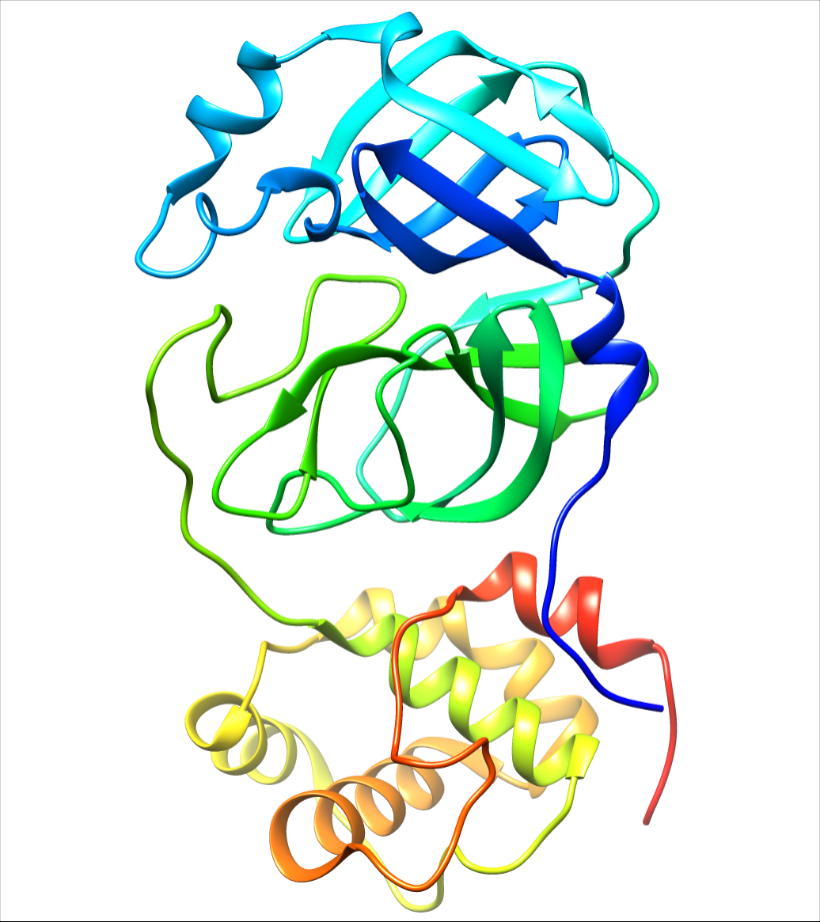
A three-dimensional cartoon representation for chain A of 2019-nCoV main protease crystal. The C-terminus is colored as red while N-terminus is colored as blue. We have used the recently released Mpro crystal with code 6LU7 [19]. We have employed UCSF chimera version 1.13.1 for image processing and rendering [20].

Coronaviruses (CoVs) are positive-sense and single-stranded RNA viruses that can cause a number of respiratory diseases in mammals [1, 2]. In the last two decades, two beta coronaviruses were responsible for the epidemic outbreak of atypical pneumonia cases. The first outbreak was caused by severe acute respiratory syndrome coronavirus (SARS-CoV) while the later epidemic was related to Middle East respiratory syndrome coronavirus (MERS-CoV) [3,4]. The global mortality rate for SARS-CoV was 9.6% and 34.4% as documented for MERS-CoV, death was the outcome of progressive acute respiratory distress syndrome or multiple organs failure [5,6]. Respiratory diseases caused by CoVs is believed to be the result of zoonotic transmission from specific animals [7]. Genomic sequencing studies suggest that the potential zoonotic reservoir of SARS-CoV is bats while camels are thought to be a probable vector or reservoir for MERS-CoV [8, 9]. In December 2019, multiple cases of unknown viral pneumonia had been reported in Wuhan, China. By that time, most of the patients were living or working near a local seafood market [10]. Genomic sequencing of patients' specimens collected from lower respiratory tract had revealed the implication of unprecedented type of coronavirus, which was later named 2019 novel coronavirus (2019-nCoV) [11]. Analysis of next generation sequencing data has showed that 2019-nCoV is similar to SARS-CoV and MERS-CoV with a genomic identity of 79% and 50% respectively [12]. According to WHO situation report of February 27, 2020, the current epidemic outbreak of pneumonia caused by 2019-nCoV has a global distribution that affects 47 countries. The confirmed cases of coronavirus disease 2019 (COVID-19) are 82,294 and most of them are living in China, the reported number of deaths is 2,804 [13]. Pneumonia cases of 2019-nCoV had clinical features very similar to those reported with SARS-CoV and MERS-CoV. COVID-19 patients were presented with fever, dry cough, dyspnea and bilateral ground-glass opacity and consolidation of chest as seen in CT images. Unlike SARS-CoV and MERS-CoV, patients with 2019-nCoV had rarely showed signs of enteric disease like diarrhea. Also, few COVID-19 patients had presented with upper respiratory tract symptoms like sore throat and rhinorrhea [11,14,15]. The transmission mode of 2019-nCoV may be similar to SARS-CoV and MERS-CoV through airborne droplets and contact with infected persons [13]. The mean incubation period is estimated to be 5 days with 95% confidence interval range of 4-7 days [16]. Like SARS-CoV, 2019-nCoV may use angiotensin-converting enzyme 2 (ACE2) as a potential receptor for host cell infectivity [17]. Currently, no antiviral therapy or vaccine is available against coronavirus infection and attempts in this trend are accelerated to combat current epidemic outbreak [7, 11]. On February 6, 2020, worldwide protein data bank has established COVID-19 coronavirus resources to facilitate target based drug design efforts against current global threat [18]. As listed in COVID-19 coronavirus resources, a crystallization team in Shanghai Technical University was able to resolve the structure of main protease for 2019-nCoV [19]. The three-dimensional representation for the monomer of 2019-nCoV main protease as deciphered from 6LU7 crystal can be seen in (Figure 1). The main protease (Mpro), also less commonly known as 3CL protease, is believed to be essential for coronaviruses replication cycle through posttranslational processing of RNA replicase machinery [21]. The Mpro is usually present as a homodimer, many coronaviruses share a significant homology regarding three-dimensional structure and amino acids sequence of this proteolytic enzyme. Therefore, Mpro represents a conserved molecular target to design a broad spectrum anti-CoV drug [22]. It is of interest to study the binding features of 1615 FDA approved drugs with the recently known 2019-nCoV main protease structure having high sequence homology with that from SARS-CoV.

## Materials and Methods:

### Superimposition and alignment analysis:

The 6LU7 crystal for 2019-nCoV main protease was superimposed and then aligned with a reference crystal of SARS-CoV main protease with code 2AMQ [23]. Both MatchMaker and Match (Align) tools embedded in UCSF chimera had been utilized to superimpose and align chain A only for these two crystals [24]. MatchMaker tool can superimpose two proteins by using residues types and/ or secondary structure information. On the other hand, Match (Align) tool can align the superimposed proteins depending on residues spatial proximities.

### Binding pockets prediction:

Although the binding site is well characterized for N3 inhibitor within many Mpro crystals [22]. We have applied DoGSiteScorer online tool to predict and describe potential binding pockets within the recently released 6LU7 crystal [25]. DoGSiteScorer tool can detect potential binding pockets within a specific protein and then rank these pockets according to their size, surface area and druggability score.

### Structure-based virtual screening:

We have used FDA approved drugs library as a resource for potential hits in our virtual screening. The FDA approved drugs library was downloaded from ZINC 15 database on February 13, 2020 [26]. By that time, the downloaded library did contain 1615 FDA approved drugs. These drugs were uploaded as 3D conformations into MCULE online drug discovery platform [27]. We have employed MCULE platform to screen these FDA approved drugs against 2019-nCoV main protease crystals. AutoDock Vina tool embedded in MCULE platform was applied to carry out an accelerated screening [28]. Mpro crystal with code 6LU7 had been downloaded from COVID-19 coronavirus resources of the worldwide protein data bank [18,19]. Before screening, we have removed both water molecules and the bounded ligand (N3 inhibitor) from 6LU7 crystal by using UCSF chimera version 1.13.1 on local machine [20]. MCULE platform had automatically added both Gasteiger charges and polar hydrogen atoms to the uploaded crystal of Mpro by using online AutoDock tools [29]. For screening protocol, we have used a binding site area of (22 * 22 * 22) Angstrom with coordinates of (X: -12, Y: 12.5, Z: 67). Otherwise, we have used the default parameters and options to screen these FDA approved drugs against the processed 6LU7 crystal. The screening output had listed and ranked these drugs according to their minimum binding energy to Mpro crystal.

### Molecular docking:

We have picked up the top ten hits from output of structure-based virtual screening process. The screening of these top hits against Mpro crystal was repeated to confirm MCULE platform results. Here, we used UCSF chimera version 1.13.1 on local machine to run the screening process [20]. UCSF chimera software provides an easily accessible interface to process both ligands and targets, the software can easily add polar hydrogen atoms and Gasteiger charges and also ignore non-standard amino acid residues. The UCSF chimera also provide a flexible way to customize AutoDock Vina tool; we have used a local Vina tool with version of 1.1.2 [28]. The molecular docking parameters used here are similar to what we have used above except that the exhaustiveness of search was increased to eight. The clean 2D and 3D conformations of the top ten drugs were prepared by using MarvinSketch version 20.1 [30]. For each hit, the ligand-target complex with least binding energy pose was saved as PDB file for further evaluation with discovery studio visualizer version 19.1.0 [31] and dynamics simulation with YASARA Dynamics version 19.12.14 [32].

### Molecular Dynamics (MD) analysis:

Molecular Dynamics (MD) simulation is an efficient method in prediction of ligand-target interactions by considering target flexibility. The atoms and molecules of the whole complex are allowed to move and interact for a specific period of time.The trajectories of these atoms and molecules are determined through Newton's equations of motion. The potential energy for interacting atoms are determined through molecular mechanics with different force fields [33].The molecular dynamics simulation of ligand-target complex with least binding energy pose was done with YASARA Dynamics [34].The protocol for MD simulation did include an optimization for the hydrogen bonding network to enhance stability of the solute, and a pKa anticipation to fine-tune the protonation states of amino acid residues at pH value of 7.4 [35]. Sodium chloride ions were added with a concentration of 0.9%, with an excess of either Na or Cl to neutralize the complex.Following steepest descent and simulated annealing minimizations to remove possible clashes, the simulation was allowed to run for 7 nanoseconds using AMBER14 force field [36] for the solute, AM1BCC [37] and GAFF2 [38] for ligands and TIP3P for water.The cutoff value was 8 Angstrom for van der Waals forces, the default parameters were used by AMBER [39]. No cutoff limit was used for electrostatic forces by employing the Particle Mesh Ewald algorithm [40]. The equations of motions were applied with a multiple timestep of 1.25 femtoseconds for bonded interactions and 2.5 femtoseconds for non-bonded interactions at a temperature of 298K and a pressure of 1 atm [32]. After evaluation of the solute root-mean-square deviation (RMSD) as a function of simulation time, the first 7 nanoseconds were considered an equilibration time and excluded from any further analysis.

## Results and discussion:

**Figure 2:**
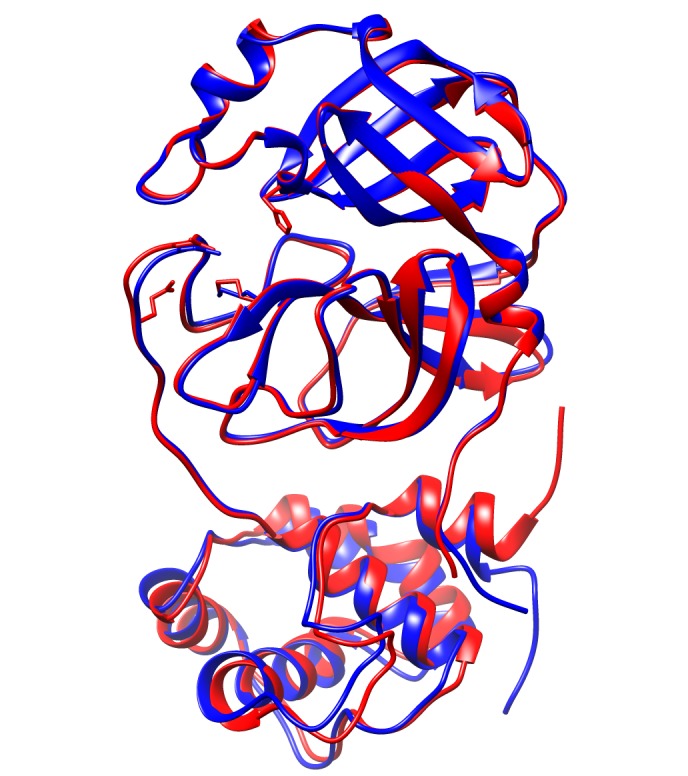
Superimposition of chain A for 2019-nCoV Mpro (blue color) with chain A for SARS-CoV Mpro (red color).

**Figure 3:**
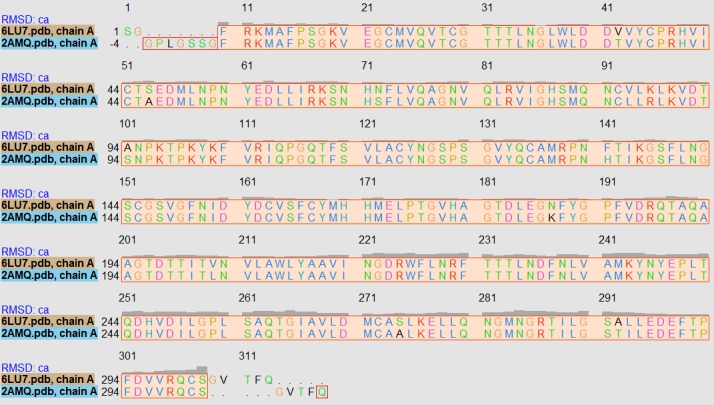
Sequence alignment output for 2019-nCoV Mpro chain A with the reference SARS-CoV Mpro chain A. The RMSD: ca represents the root-mean-square deviation for variation in each column.

**Figure 4:**
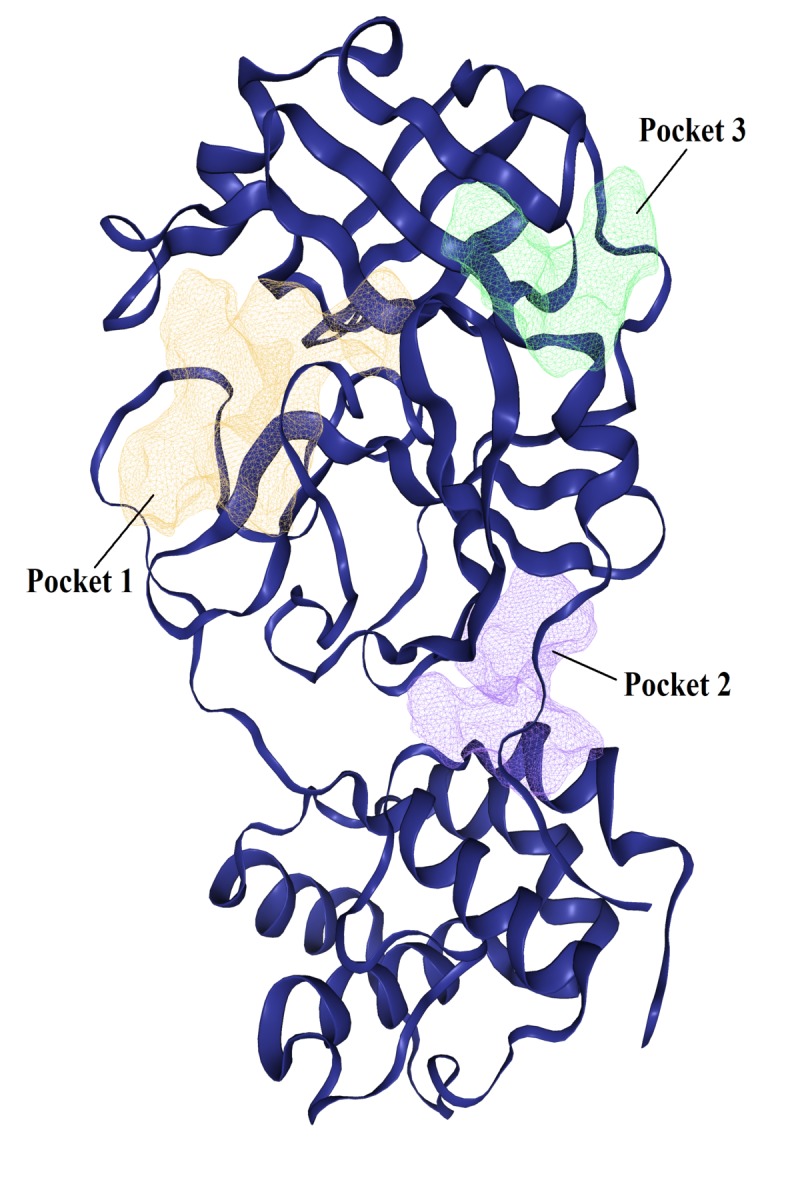
Predicted binding pockets within chain A of 2019-nCoV main protease crystal.

**Figure 5:**
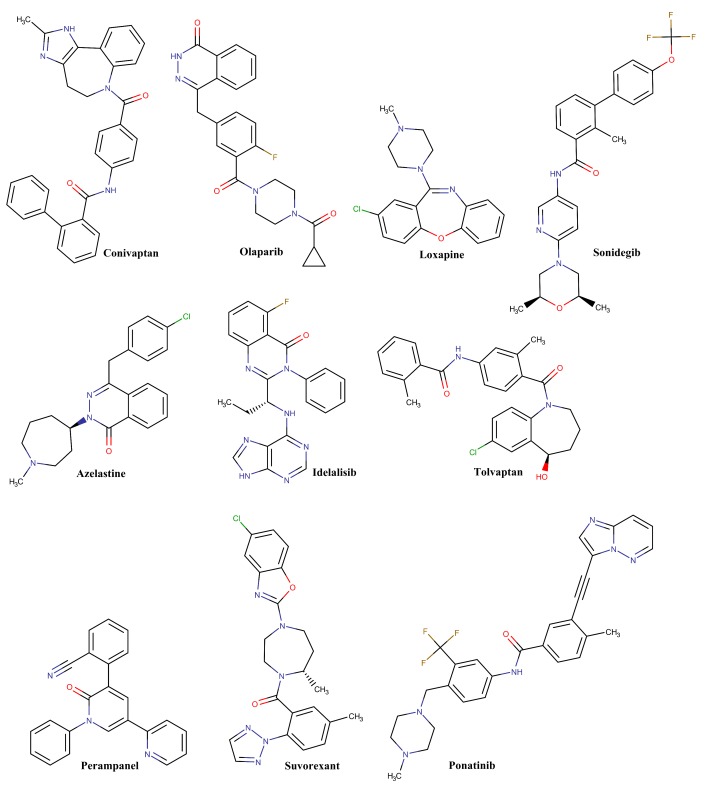
Chemical structures for the top ten drugs with least binding energy as screened virtually against 2019-nCoV Mpro crystal.

**Figure 6:**
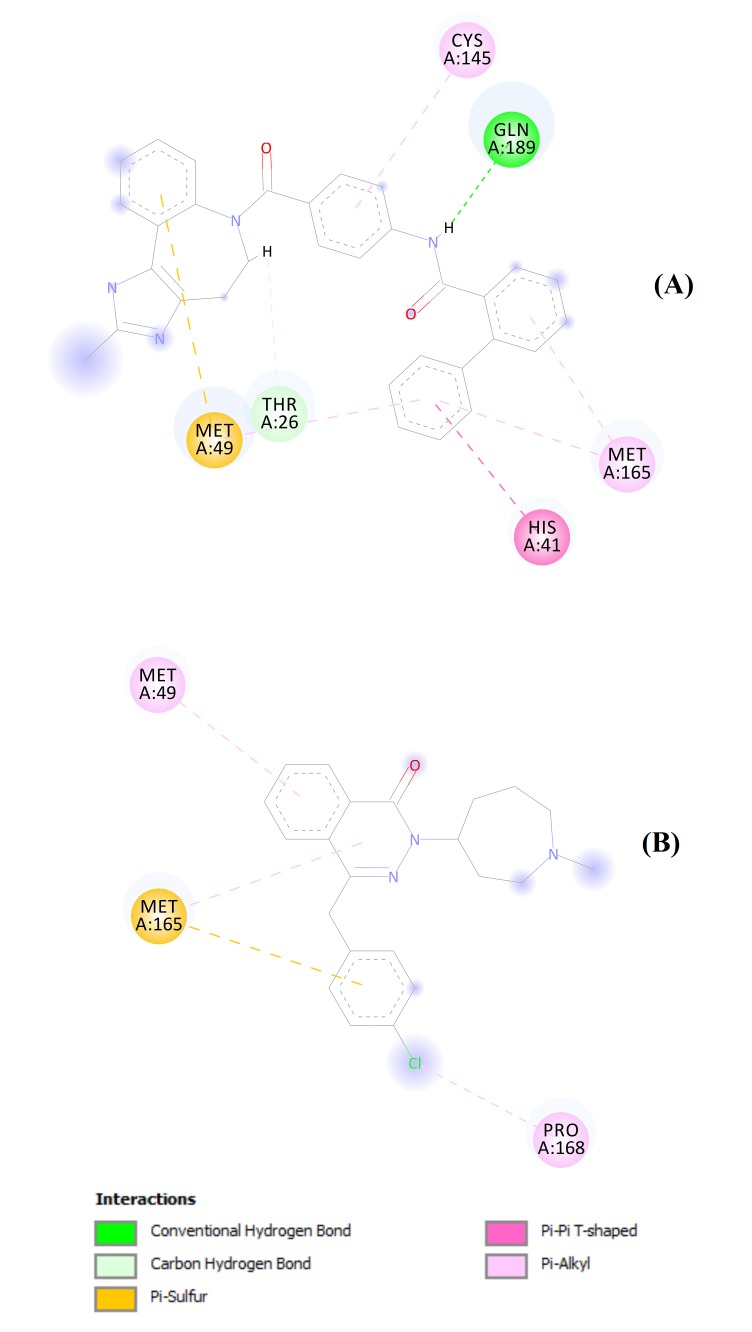
Two-dimensional representation for docking of (A) Conivaptan and (B) Azelastine against 2019-nCoV main protease crystal. The colored discs represent active site residues, while dashed lines refer to interaction bonds.

**Figure 7:**
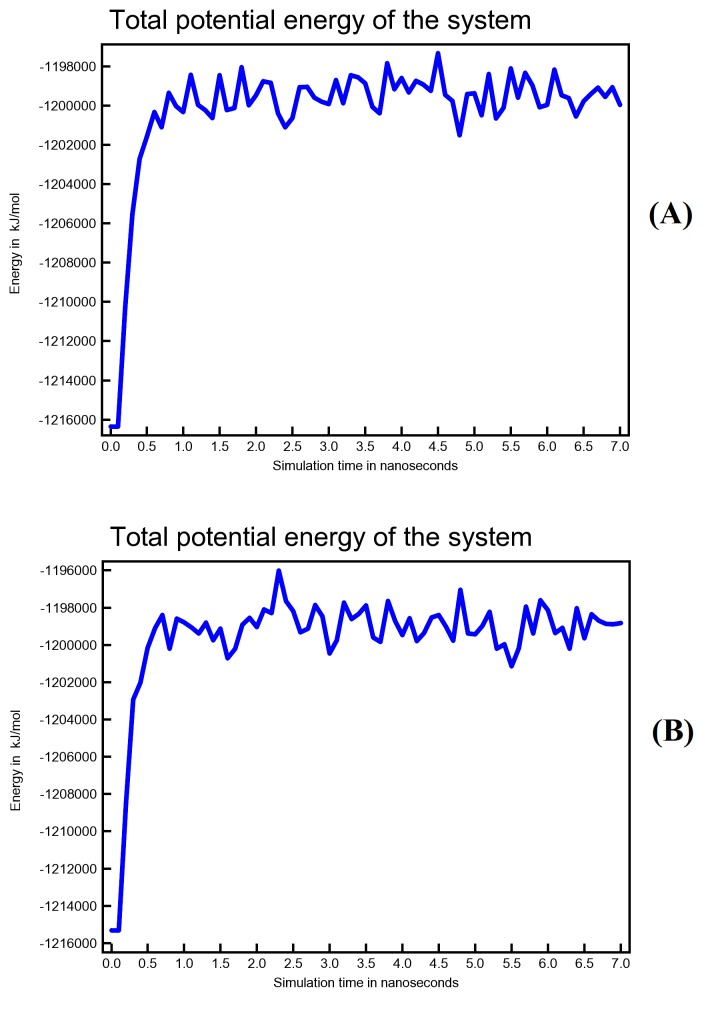
Potential energy versus simulation time plot for (A) Conivaptan-Mpro complex and (B) Azelastine-Mpro complex. Energy is expressed as KJ/ mol.

**Figure 8:**
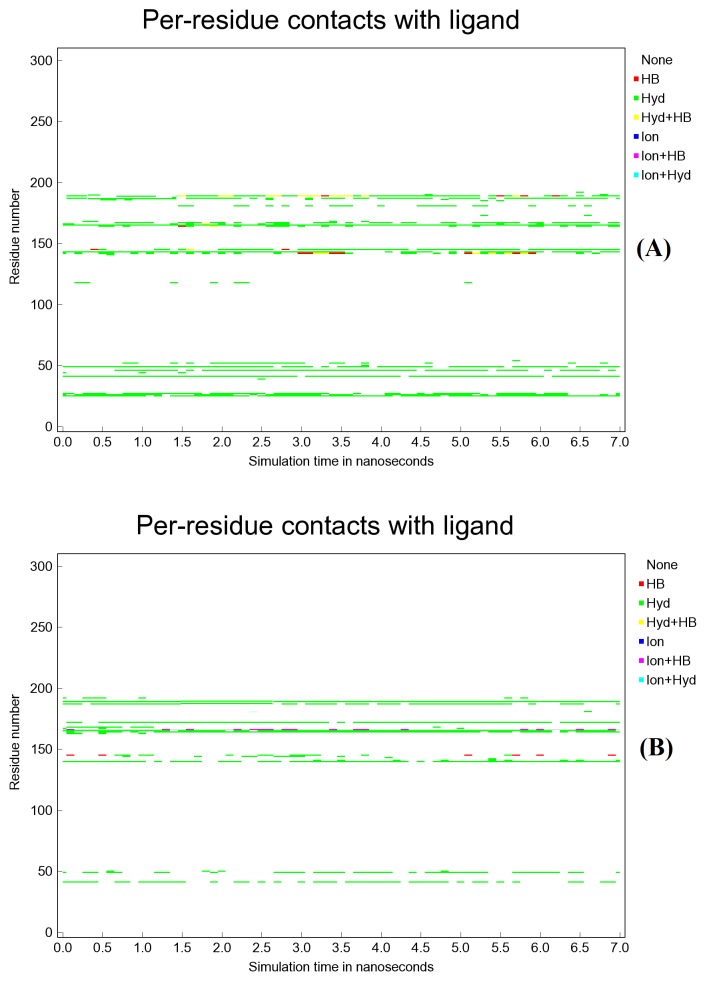
Interactions plot between Mpro active site residues and (A) Conivaptan or (B) Azelastine during 7 nanoseconds period of simulation.

**Figure 9:**
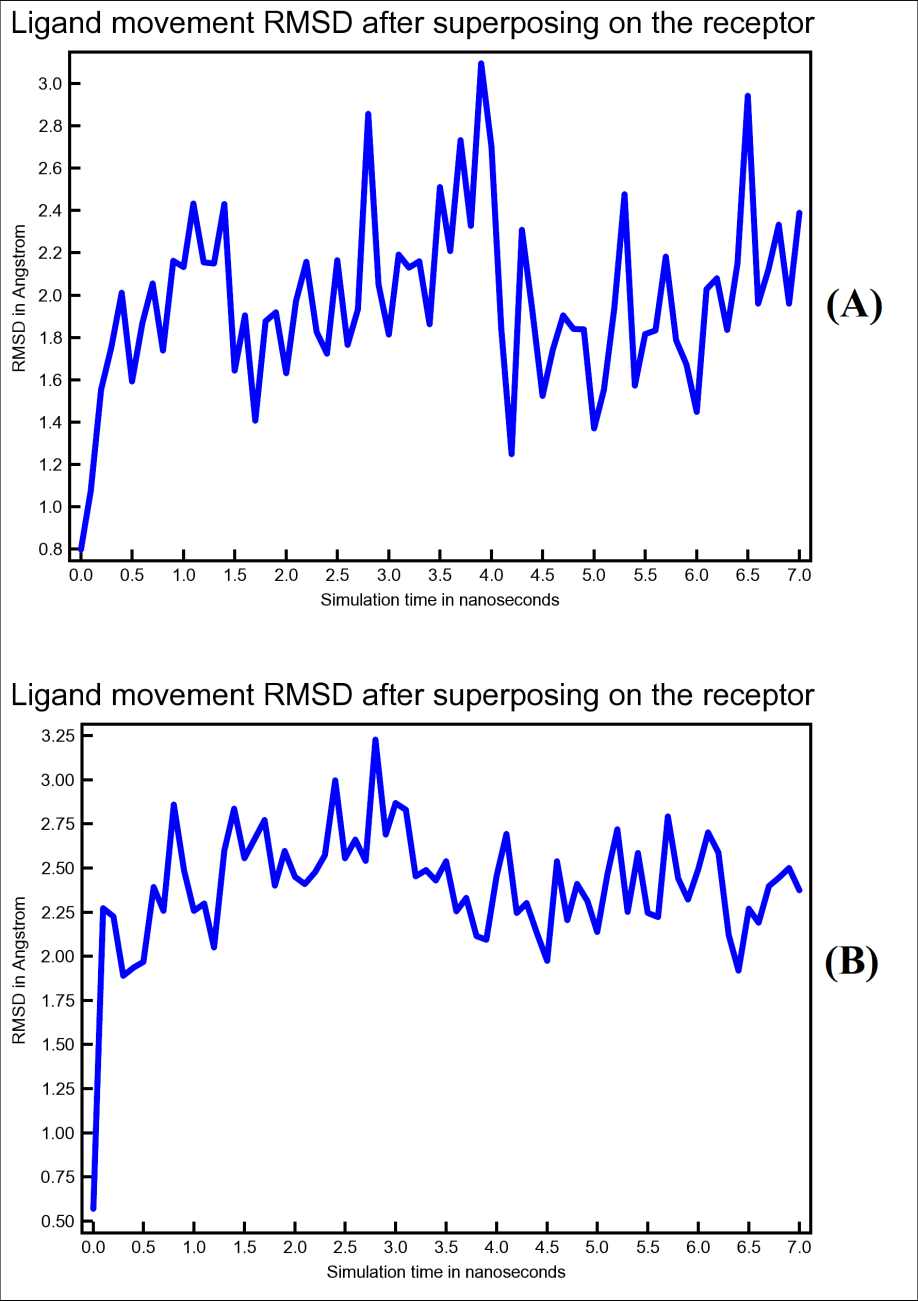
Ligand movement RMSD as a function of simulation period. Plot (A) is for Conivaptan movement while plot (B) is for Azelastine movement.

**Figure 10:**
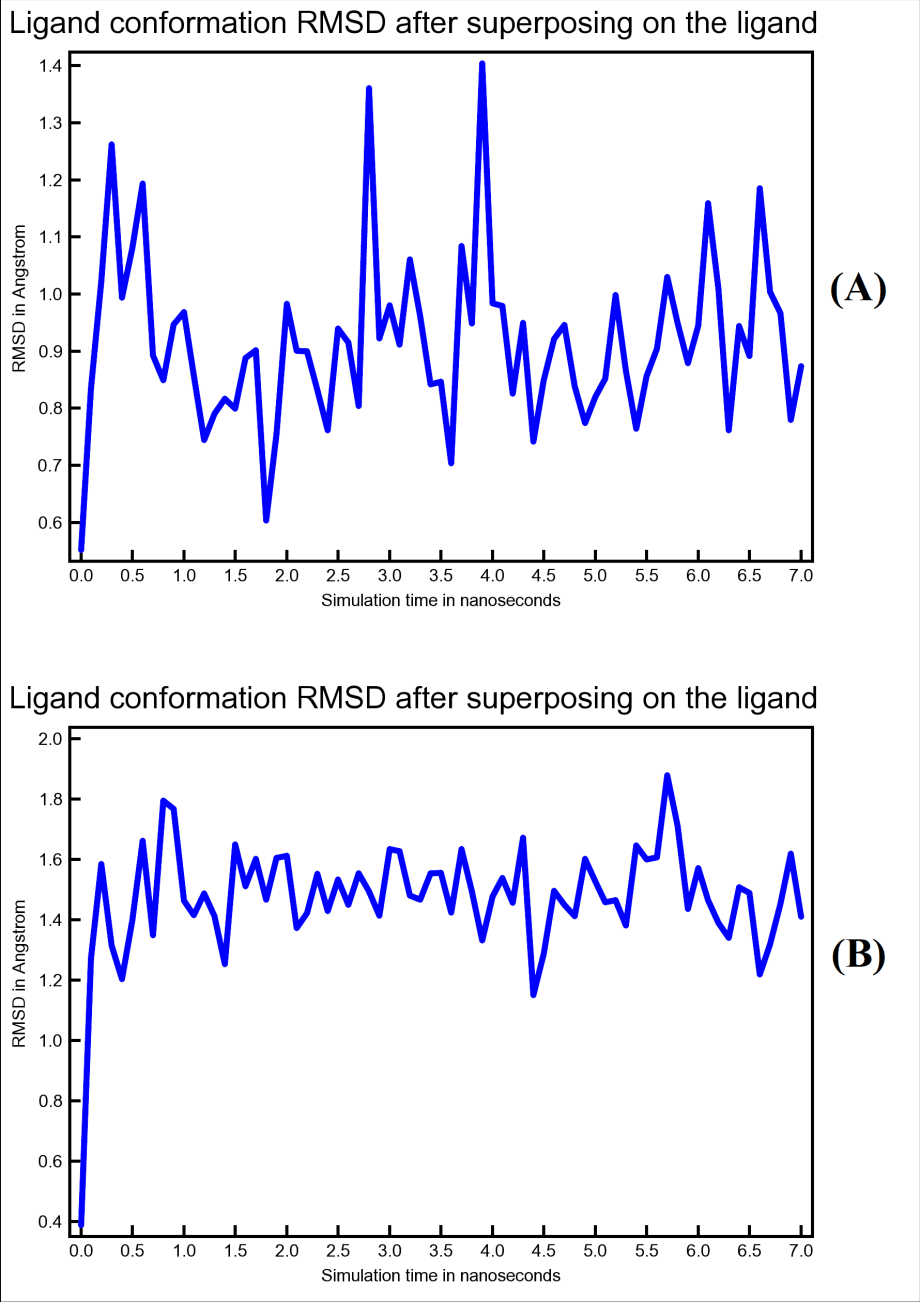
Ligand conformation RMSD as a function of simulation period. Plot (A) is for Conivaptan conformational changes while plot (B) is for Azelastine conformational changes.

By using 2AMQ crystal for SARS-CoV Mpro as a reference, we were able to successfully align and superimpose 6LU7 crystal for 2019-nCoV main protease. According to (Figure 2), both 6LU7 and2AMQ crystals are well superimposed. The only significant overlap deviation can be noticed in the C-terminus region for these two crystals. For simplicity of illustration, only chain A was considered for alignment and superimposition. These two superimposed crystals were then aligned according to residues spatial proximities.(Figure 3) reports sequence alignment output for 2019-nCoV Mpro and SARS-CoV Mpro. These two crystals are very well aligned with identity percentage of 93.79. These results fall in favor of previous studies indicating that the main protease enzyme is highly conserved in many coronavirus members, therefore this proteolytic enzyme may be used in structure-based screening studies to design a broad spectrum anti-CoV drug [22,23]. Then by employing DoGSiteScorer grid-based tool [25], we were able to detect several potential binding pockets within chain A of 2019-nCoV main protease crystal. Here, we have reported only the first three pockets in (Table 1), these binding pockets are ranked according to their size, surface area and druggability score. The first pocket is the preferred binding site for N3 inhibitor as reported in many crystallization studies of CoVs Mpro [19,22,23]. The location of pocket one was used as coordinates for our grid box setup and docking analysis. These predicted binding pockets within 6LU7 crystal are also well illustrated in (Figure 4). The chemical and clinical characteristics for the top ten hits with the least binding energy (docking score) are listed in (Table 2). These FDA approved drugs were ranked according to their minimum binding energy to 2019-nCoV main protease. According to this table, four of these drugs are anticancer agents. Due to safety concerns, they have been neglected from further analysis in this study. We also ignored Perampanel and Loxapine from our consideration due to their significant central nervous system effect. We have chosen both Conivaptan and Azelastine for further molecular docking and dynamics analysis due to their relative safety [41] and thereby they may be repurposed. Molecular dynamics (MD) analysis is computationally demanding process; therefore we have focused only on Conivaptan and Azelastine to save our limited computational power. The chemical structures for these ten drugs are shown in (Figure 5). Molecular docking images show the predominant involvement of different hydrophobic interactions between ligand (Conivaptan or Azelastine) and amino acid residues of 2019-nCoV main protease. The only exception is the formation of a hydrogen bond between glutamine 189 residue of Mpro and Conivaptan. The two-dimensional image for docking analysis of Conivaptan and Azelastine against Mpro crystal is shown in (Figure 6). It is evident that Conivaptan has more interactions with Mpro active site than does the Azelastine. The ligand-protein complex with least binding energy pose was saved as PDB file for MD simulation. According to potential energy plot in (Figure 7), both Conivaptan-Mpro complex and Azelastine-Mpro complex were stable during equilibrium phase. Based on MD analysis report, Conivaptan has more interactions with Mpro active site residues than does Azelastine during simulation period. Most of these interaction bonds are hydrophobic. These results can be seen in (Figure 8), which fall in agreement with findings of docking studies. Ligand proximity to active site residues of the target may indicate stronger binding. In this regard, both Conivaptan and Azelastine were able to maintain low ligand movement root-mean-square deviation (RMSD) that didn't exceed 3.25 Angstrom as seen in (Figure 9). Superposing the receptor on its reference structure generated this plot. Finally, the analysis of molecular dynamics has shown that Conivaptan may undergo more conformational changes as compared to Azelastine during simulation time. (Figure 10) shows ligand conformation root-mean-square deviation (RMSD) by superposing the ligand on its reference structure.

## Conclusions:

We report the optimal binding features of Conivaptan and Azelastine with the main protease protein target from 2019-nCoV using molecular docking and simulation studies for further consideration.

## Figures and Tables

**Table 1:** Binding pockets prediction for chain A of 2019-nCoV main protease crystal.

Pocket No.	Volume (A^3^)	Surface area (A^2^)	Druggability score
1	702.27	842.81	0.77
2	374.59	757.16	0.74
3	330.18	518.79	0.56

**Table 2:** Chemical and clinical characteristics of the top ten drugs as screened virtually against 2019-nCoV main protease. These drugs were ranked according to their minimum binding energy to main protease crystal of 2019-nCoV.

No.	Generic name	Molecular formula	Binding energy (Kcal/mol)	Indication [41]	Legal status [41]
1	Perampanel	C23H15N3O	-8.8	Epilepsy	POM
2	Conivaptan	C32H26N4O2	-8.6	Hyponatremia	POM
3	Sonidegib	C26H26F3N3O3	-8.5	Basal-cell carcinoma	POM
4	Azelastine	C22H24ClN3O	-8.4	Allergy	POM
5	Idelalisib	C22H18FN7O	-8.1	Leukemia and	POM
				lymphoma	
6	Suvorexant	C23H23ClN6O2	-8.1	Insomnia	POM
7	Olaparib	C24H23FN4O3	-8	Ovarian, breast and	POM
				pancreatic cancers	
8	Ponatinib	C29H27F3N6O	-8	Leukemia	POM
9	Loxapine	C18H18ClN3O	-7.6	Schizophrenia	POM
10	Tolvaptan	C26H25ClN2O3	-7.5	Hyponatremia	POM
